# Chiari Malformation Type I With Concurrent Bilateral Optic Disc Drusen: Is Follow-up Necessary?

**DOI:** 10.7759/cureus.63937

**Published:** 2024-07-06

**Authors:** Maryam I Alkhayat, Hana A Almuhawas, Safaa S Almazrouei, Sameh E Soliman

**Affiliations:** 1 Pediatric Ophthalmology, King Abdullah Specialist Children's Hospital, Riyadh, SAU; 2 Ophthalmology, Salmaniya Medical Complex, Manama, BHR; 3 Radiology, King Abdulaziz Medical City Riyadh, Riyadh, SAU; 4 Ophthalmology, Faculty of Medicine, Alexandria University, Alexandria, EGY

**Keywords:** multimodal ophthalmic imaging, optical coherence tomography, pseudopapilledema, papilledema, optic disc drusen, chiari malformation

## Abstract

Pseudopapilledema caused by optic disc drusen (ODD) mimics the appearance of papilledema and usually presents as a diagnostic challenge. A young boy with known Chiari malformation type 1 (CM-1) was referred to the pediatric ophthalmology clinic for eye assessment to exclude papilledema due to elevated intracranial pressure (ICP). Despite the ophthalmic examination revealing bilateral optic disc elevation, multimodal imaging techniques such as fundus autofluorescence, optical coherence tomography (OCT), and B-scan ultrasonography are recommended to confirm the distinction between bilateral ODD causing pseudopapilledema and papilledema secondary to elevated ICP.

Accidental coexistent papilledema mimickers like ODD need to be considered in patients with CM-1 before making a diagnosis of papilledema to avoid unnecessary invasive procedures. There was no evidence that the presence of ODD excludes the possibility of future optic nerve head changes due to elevated ICP. The multidisciplinary consensus decided on annual ophthalmology follow-ups using multimodal imaging to detect any subtle optic nerve head changes.

## Introduction

Chiari malformation type 1 (CM-1) is a congenital disorder characterized by inferior displacement of the cerebellar tonsils through the foramen magnum that may compress the brainstem, cranial nerves, cerebellum, and/or spinal cord [1]. Ophthalmic evaluation may identify oculomotor/abducent nerve paresis, any vergence movement abnormalities, or optic nerve head swelling (papilledema).

Papilledema though rare can be the only neurological presentation of elevated intracranial pressure (ICP). This serious manifestation usually requires surgical suboccipital decompression and cannot be confirmed by cerebrospinal fluid (CSF) studies through lumbar puncture due to the foramen magnum crowding. This highlights the value of ophthalmic assessment in such cases [1,2].

Pseudopapilledema is different from papilledema in terms of its clinical implications. Pseudopapilledema is characterized by an elevated appearance of the nerve head without edema of the nerve fiber layer. Optic disc drusen (ODD), congenital disc defects, myelinated nerve fibers, and peripapillary masses such as astrocytic hamartomas are among the various optic disc abnormalities that can create the appearance of pseudopapilledema [3,4].

We present a rare CM-1 case that presented to confirm/rule out papilledema and was found to have bilateral ODD causing papilledema-like picture or pseudopapilledema.

## Case presentation

**Figure 1:**
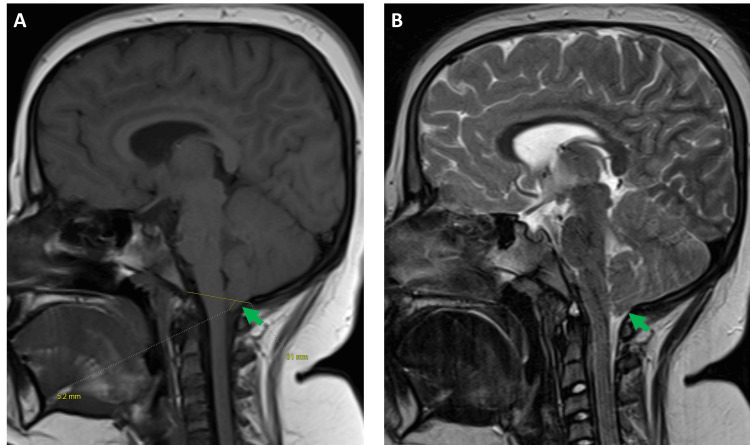
Magnetic resonance imaging (MRI) (A) Sagittal T1 and (B) sagittal T2 images demonstrate cerebral tonsils migrating 5.2 mm through the foramen magnum, causing indentation upon the cervicomedullary junction along with effacement of adjacent subarachnoid space (green arrow). No evidence of syringomyelia was found.

A young boy known to have CM-1 (Figure 1) was referred to our pediatric ophthalmology clinic for a baseline assessment. The child has been followed up elsewhere for eight years and has never been assessed by an ophthalmologist. On examination, the best-corrected visual acuity was 20/20 in each eye. Orthoptic and slit lamp biomicroscopic assessments were within normal range. Optic nerve function tests, including color vision, pupillary light reflex, and visual fields were normal. Cycloplegic refraction showed +1.0 in each eye.

**Figure 2:**
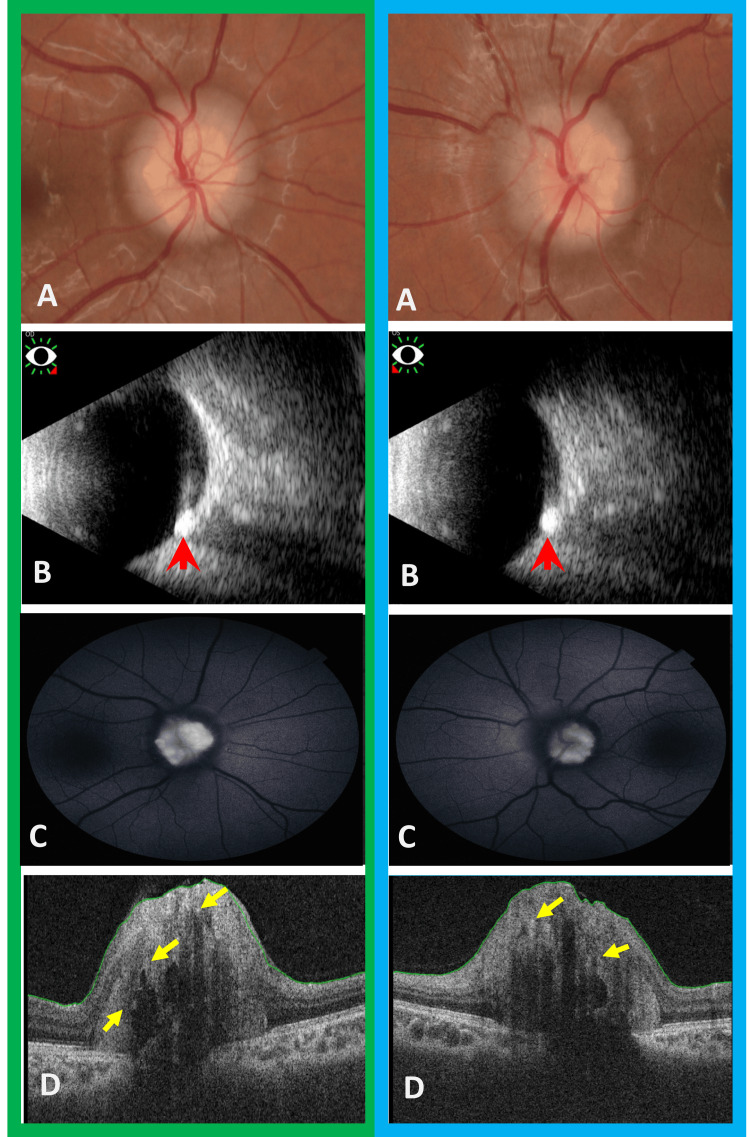
Composite figure showing multimodal assessment of optic nerve head (A) Color fundus photograph showed that both optic discs are "lumpy bumpy" in appearance with highly refractile bodies seen extruding from the disc margins. (B) B-scan ocular ultrasound showed calcified drusen seen as hyperechoic lesions with posterior shadowing (red arrowhead). (C) Fundus autofluorescence photograph showed highly refractile exposed optic disc drusen. (D) Optical coherence tomography (OCT) showed that buried optic disc drusen are characterized as oval hyporeflective voids with overlying scattered hyperreflective dots (yellow arrows). The green boxes indicate the right eye, while the blue boxes indicate the left eye.

A dilated fundus examination showed bilateral elevation of the optic nerve head with blurry margins and obliterated cups (Figure 2, Panel a). This clinical picture raised the concern for possible papilledema. However, there was no hyperemia, retinal vessel tortuosity, or obscuration. There were absent peripapillary hemorrhages, exudates, watermarks (Paton folds), and choroidal folds. Prolonged optic nerve head monitoring showed preserved spontaneous venous pulsations.

B-scan ultrasonography, autofluorescence, and optical coherence tomography (OCT) were performed, which showed hyperechoic, hyper-autofluorescent, hyperreflective structure within the optic nerve head, confirming the diagnosis of bilateral ODD (Figure 2, Panels b-d). Automated perimetry was unreliable with high false positives repeated twice.

**Figure 3:**
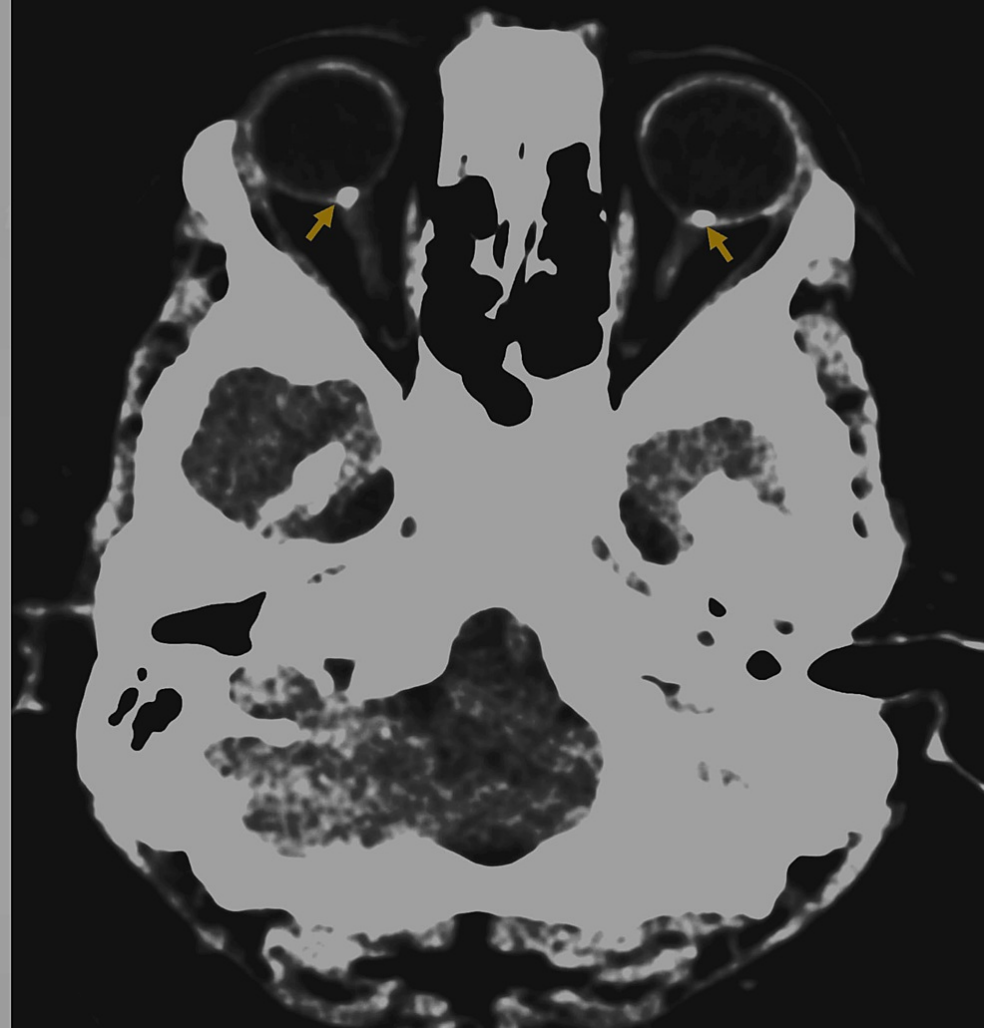
Computed tomographic scan of head and orbit showing bilateral optic nerve head calcification (yellow arrow) consistent with optic disc drusen

Despite the ophthalmology confidence in ODD diagnosis, the neurosurgical team insisted on performing a computed tomography (CT) scan (Figure 3). The diagnosis of bilateral ODD was established with the exclusion of elevated ICP at the current status. The multidisciplinary consensus was to continue the annual comprehensive ophthalmology evaluation with multimodal optic nerve head imaging to detect any change that might raise the suspicion of elevated ICP.

## Discussion

The diagnosis of papilledema in cases of CM-1 needs to be thoroughly verified. Lumbar puncture for CSF opening pressure measurement is contraindicated as it carries the risk of medullary coning with foramen magnum crowding, which is common with CM-1. The ophthalmologist carries the higher burden to confirm/rule out this diagnosis. Clinical assessment is usually sufficient in cases of well-established papilledema where peripapillary hemorrhages, soft exudates, and choroidal folds associate the optic disc elevation. OCT is vital to differentiate between papilledema and pseudopapilledema. Papilledema shows a thicker peripapillary retinal nerve fiber layer (RNFL) in all quadrants, and nasal RNFL thickness has the highest diagnostic relevance in diagnosing papilledema. Bekerman et al. [1] suggested that optic nerve sheath diameter assessment on MRI is a more reliable diagnostic tool than clinical ophthalmologic assessment to reflect elevated ICP in CM-1 cases.

ODD is a common cause of pseudopapilledema that has been reported in 1%-2.4% of the general population [5-7]. Calcium/hyaline deposit between the lamina cribrosa and Bruch’s membrane (deep/buried ODD) causes optic disc elevation. The ODD might become more superficial and thus visible with age. Multimodal imaging is necessary to confirm ODD diagnosis [5]. The ODD appears as a hyperechoic nodule in B-scan ultrasonography with absent fluid signs (crescent/donut sign). OCT shows corresponding hyperreflective areas in the optic nerve head. Autofluorescence shows hyperfluorescent lesions with irregular borders within the optic nerve head [5,8,9].

Sarac et al. [2] reported a similar case with ODD and CM-1 in a 32-year-old patient. Unfortunately, they did not provide a follow-up plan for their case. The presence of ODD does not exclude the possibility of concurrent elevated ICP. Genizi et al. [10] reported that around 15% of children with pseudotumor cerebri had concurrent ODD. Furthermore, in certain diseases such as CM-1, elevated ICP develops during the course of the disease, potentially leading to papilledema in conjunction with pre-existing ODD. Papilledema is rare in CM-1 cases but can occur in around 2% of cases [2].

Our case involved a young teenager, prompting the neurosurgical team to inquire about the need for follow-up. It is noteworthy that most published reports show that papilledema typically manifests at an older age, generally between the third and sixth decades. We advised annual follow-up using multimodal imaging to closely monitor the optic nerve head and ODD and to detect any signs of progression. However, if symptoms such as headaches, visual disturbances, or vomiting arise, an earlier evaluation would be warranted. It is worth mentioning that CT is not usually recommended in the diagnosis of ODD to detect calcifications. Due to the high concern from the neurosurgical team in our case despite the ophthalmologist's assurances, CT was performed. Fortunately, ODD calcifications revealed by the CT helped convince the neurosurgical team of the ODD diagnosis.

## Conclusions

In CM-1, any elevation in the optic disc head needs a thorough evaluation by an expert ophthalmologist to confirm or rule out papilledema and differentiate it from common causes of pseudopapilledema as ODD. Proper assessment and evaluation are required to avoid unnecessary invasive procedures. However, if ODD is diagnosed in association with conditions such as CM-1 with a known possibility of papilledema along its course, routine follow-up is recommended via clinical optic disc assessment, autofluorescence, and OCT.
